# Epstein-Barr Virus Provides a New Paradigm: A Requirement for the Immediate Inhibition of Apoptosis

**DOI:** 10.1371/journal.pbio.0030404

**Published:** 2005-11-15

**Authors:** Markus Altmann, Wolfgang Hammerschmidt

**Affiliations:** **1**Department of Gene Vectors, GSF-National Research Center for Environment and Health, München, Germany; University of Wisconsin at MadisonUnited States of America

## Abstract

DNA viruses such as herpesviruses are known to encode homologs of cellular antiapoptotic viral Bcl-2 proteins (vBcl-2s), which protect the virus from apoptosis in its host cell during virus synthesis. Epstein-Barr virus (EBV), a human tumor virus and a prominent member of γ-herpesviruses, infects primary resting B lymphocytes to establish a latent infection and yield proliferating, growth-transformed B cells in vitro. In these cells, 11 viral genes that contribute to cellular transformation are consistently expressed. EBV also encodes two *vBcl-2* genes whose roles are unclear. Here we show that the genetic inactivation of both *vBcl-2* genes disabled EBV's ability to transform primary resting B lymphocytes. Primary B cells infected with a *vBcl-2*-negative virus did not enter the cell cycle and died of immediate apoptosis. Apoptosis was abrogated in infected cells in which *vBcl-2* genes were maximally expressed within the first 24 h postinfection. During latent infection, however, the expression of *vBcl-2* genes became undetectable. Thus, both vBcl-2 homologs are essential for initial cellular transformation but become dispensable once a latent infection is established. Because long-lived, latently infected memory B cells and EBV-associated B-cell lymphomas are derived from EBV-infected proapoptotic germinal center B cells, we conclude that *vBcl-2* genes are essential for the initial evasion of apoptosis in cells in vivo in which the virus establishes a latent infection or causes cellular transformation or both.

## Abstract 2

The transformation of resting B-lymphocytes by a human tumor virus is shown to require vBcl-2 genes, which abrogate host cell apoptosis. These genes are not required once latent infection is established.

## Introduction

Apoptosis is a mechanism used by the infected cell as part of its antiviral response; cells can commit suicide as a direct response to viral infection prior to viral maturation. Therefore, regulating apoptosis during infection is a high priority for many viruses that frequently encode Bcl-2-like mechanisms to do so [1]. Cellular Bcl-2 was originally discovered as an oncogenic protein in follicular lymphomas and up to now many Bcl-2 family members have been identified that share distinct domains of high sequence homology. Bcl-2 family members include apoptosis-inhibitory proteins, such as Bcl-2 and Bcl-X_L_, or those that mediate proapoptotic functions exemplified by Bax. All viral Bcl-2 (vBcl-2) homologs identified so far block apoptosis. The major role of vBcl-2 proteins is therefore to prevent premature death of the host cell during virus production, which would otherwise reduce the amount of progeny virus [2,3]. Adenovirus E1B 19K, for example, is a minimal version of the cellular Bcl-2 prototype, which prevents premature cell death during productive infection. Bcl-2 and E1B 19K can functionally substitute for each other in the suppression of apoptosis during virion synthesis and oncogenic transformation [1,3]. While members of the γ-herpesvirus family carry one *vBcl-2* gene in general, Epstein-Barr virus (EBV) encodes two *Bcl-2* homologs, *BALF1* and *BHRF1.* The BHRF1 protein clearly resembles Bcl-2 in its antiapoptotic function during in vitro assays [4], while the role of BALF1 is controversial [5,6]; however, no function has been assigned to either BALF1 or BHRF1 in the context of viral infection [1,7]. Interestingly, vBcl-2 homologs have not been recognized to contribute to viral latent infection directly [2,8,9], probably because viral latency can only be studied with very few viruses. In addition, functions involved in viral “fitness” often overlap with specific functions, which contribute directly to initiation and maintenance of viral latency in vivo [10–12].

EBV provides a unique in vitro model, which permits the dissection of viral contributions to latent infection. EBV can infect all cells of the B-cell lineage, but its main targets in vivo are naïve B cells and B cells that undergo affinity maturation during a germinal center reaction to establish long-term latent infections in memory B cells [13,14]. In vivo and in vitro EBV's latent state is characterized by the absence of virus synthesis and maintenance of the viral genome as plasmids in the infected cell. In vitro, EBV infects resting human B lymphocytes and transforms them into lymphoblastoid cell lines (LCLs), a process that is termed growth transformation and a hallmark of this virus. In LCLs, 11 so-called latent genes are consistently expressed. These are the EBV nuclear antigens EBNA1, EBNA2, EBNA-LP, EBNA3A, -B, -C, and the latent membrane proteins LMP1, LMP2A, and -B, and two noncoding RNAs [15]. Genetic and biochemical experiments demonstrate that several of these viral proteins directly contribute to growth transformation and latency of infected cells in vitro and in vivo, although only a subset of these 11 genes are expressed in the human host [16]. Typically, latent genes of EBV mimic cellular functions. For example, the viral proteins LMP1 and LMP2A target physiological signaling pathways, which are engaged by the B-cell and CD40 receptors upon contact with antigen and T helper cells, respectively. LMP1 and LMP2A are also expressed in EBV-positive B-cell lymphomas such as Hodgkin's disease and posttransplant lymphomas [13], which are two of several malignancies with which EBV has been associated [16,17]. LMP1 indirectly inhibits apoptosis by upregulating several cellular antiapoptotic genes [18], presumably through the induction of the NF-κB pathway [19–21]. LMP1 promotes cell proliferation [18,22] and scores as an oncogene in transgenic animals [23]. In LCLs, but not in EBV-associated tumors, LMP1 and LMP2A are transcriptionally upregulated by EBNA2, which is the first latent viral gene expressed after infection of B cells in vitro. EBNA2 is the key viral factor, which is essential for in vitro growth transformation of resting primary B cells [24,25], along with LMP1 [26], EBNA1 [15], EBNA3A, and -C [27]. This minimal set of five latent EBV genes has been regarded as sufficient to yield growth-transformed LCLs in vitro [15], although the requirements for EBNA3A, EBNA1, and LMP1 appear to be less stringent [28–30]. None of the known latent gene products of EBV directly regulate apoptosis, and little is known about viral genes expressed very early in primary B cells upon viral infection.

We analyzed the two *vBcl-2* genes of EBV—*BHRF1* and *BALF1*—and demonstrate that their gene products in addition to the known latent EBV genes are essential for the process of B-cell transformation. The *vBcl-2* homologs are maximally expressed initially after infection but are neither expressed nor required once a latent infection is established. The very early expression of *vBcl-2* genes prevents EBV-infected B cells from undergoing spontaneous programmed cell death and is mandatory to establish a latent infection and cause cellular transformation. We conclude that EBV employs a biphasic control—characterized by viral genes expressed transiently and those that are expressed stably—to initiate and maintain a latent infection.

## Results

### *vBcl-2* Genes Are Essential for Growth Transformation of Resting B Cells but Dispensable for Proliferating B Blasts

In order to gain insight into the function of *BALF1* and *BHRF1,* we constructed several viral mutants, which carry singly or dually inactivated alleles of *BALF1* and *BHRF1* (Figure 1A and 1B). Mutant virus stocks were generated as described [31]. Surprisingly, virus production was not impaired even in the case of the dually inactivated *vBcl-2* mutants, and transcomplementation of either *BALF1* or *BHRF1* in virus-producing cells did not yield higher titers (data not shown). Virus stocks were quantified by infecting Raji cells, an established B-cell line [29]. Because our recombinant EBVs encode green fluorescence protein (GFP), we could measure the concentration of GFP-transducing virions as “green Raji units” (GRUs), which were in the range of 10^4^–10^5^/ml GRUs similar to wild-type 2089 EBV stocks [32]. Primary B cells were infected with serial virus dilutions to determine the number of GRUs required statistically to give rise to clonal LCLs [33]. Two GRUs were sufficient in the case of 2089 wild-type EBV, and the single *BALF1^–^* or *BHRF1^–^* mutants were equally capable of yielding clonal LCLs but at a slightly higher virus dose (Figure 1B–1D). Two independent viral mutants with inactivated *vBcl-2* genes failed to generate LCLs, even at a dose of 10^4^ GRUs per well (Figure 1C and 1D). Reconstruction of the *BALF1* allele in the revertant *BALF1^+^/BHRF1^−^* virus reconstituted EBV's capacity to growth transform human B lymphocytes (Figure 1C and 1D).

**Figure 1:**
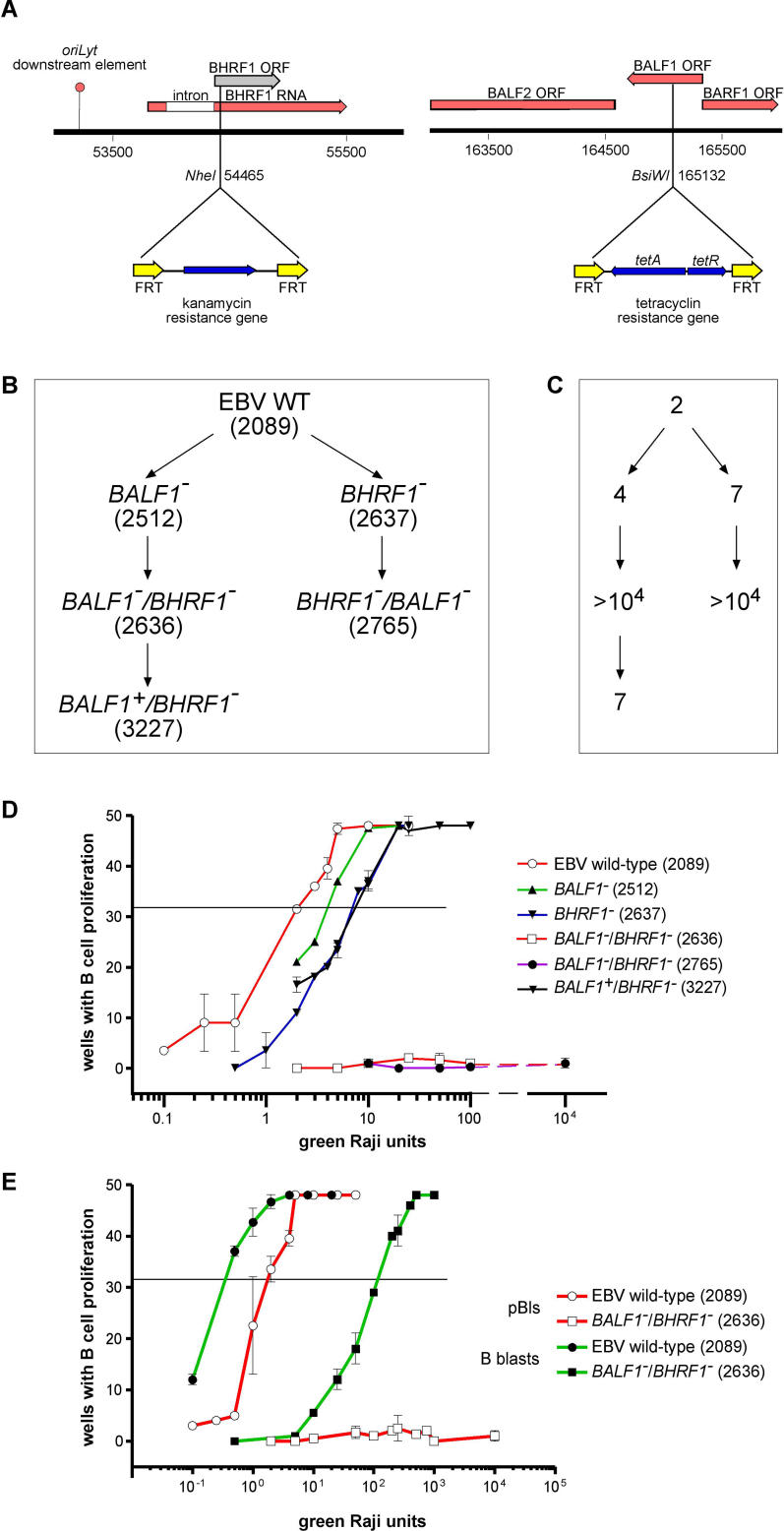
Construction and Genealogy of EBV Mutants and Their Efficiency of B-Cell Growth Transformation (A) Construction of the loss of function mutation in *BALF1* and *BHRF1.* (B) Wild-type (WT) 2089 EBV is based on the genome of the EBV strain B95.8. The *BALF1* and *BHRF1* genes were inactivated through insertional mutagenesis with antibiotic resistance genes. Two *vBcl-2* mutants 2636 and 2765 were constructed independently on the basis of the singly inactivated viruses, and the *BALF1^−^*/*BHRF1^−^* mutant 2636 was reverted to *BALF1^+^*/*BHRF1^−^.* (C) The numbers indicate the number of GRUs of the virus stocks, which were required to yield one clonal LCL for each virus noted in (B). (D) Efficiency of B-cell growth transformation. The different singly and dually *vBcl-2^−^* EBV virus mutants were compared with wild-type 2089 EBV for their efficiencies to growth transform primary B cells in limiting dilution assays. Forty-eight wells with 10^5^ target B cells per well were infected with each virus dilution, and wells with proliferating cells were recorded 6 wk p.i. The data are graphed to identify the number of GRUs of the different virus isolates required to yield one LCL. The horizontal line at 30 wells positive for 48 wells plated identifies for a Poisson distribution the required number of GRUs shown on the x-axis. Infection of primary B cells with the *vBcl-2^−^* mutants 2636 and 2765 did not yield stable LCLs even with up to 10^4^ GRUs per 10^5^ cells per well. (E) Comparison of the efficiency of wild-type 2089 EBV and the *BALF1^−^*/*BHRF1^−^* mutant 2636 to yield stable, clonal LCLs in limiting dilution assays with primary B cells or activated B blasts as target cells. Activated B blasts readily gave rise to LCL clones with the *BALF1^−^*/*BHRF1^−^* mutant 2636 in contrast to the situation in (D), although about 200 GRUs were statistically required to establish clonal lines, which all could be further expanded and characterized (data not shown).

We determined whether infection of preactivated human B cells might overcome the failure of the *vBcl-2^−^* mutants to establish growth transformed LCLs from resting primary B cells. B blasts, which can be generated from resting primary B cells by cocultivation on CD40 ligand-expressing feeder cells in conjunction with IL-4, proliferate in vitro and express cellular antiapoptotic Bcl-2 family members, which are upregulated through the CD40 and JAK/Stat-induced signaling pathways [34,35]. The continued proliferation of B blasts is totally dependent on both activating stimuli. Upon removal of either CD40L or IL-4 signals, the B blasts cease to proliferate immediately and undergo apoptosis within 10 d (data not shown). Limiting dilution assays with activated B blasts as target cells and serially diluted *BALF1^−^*/*BHRF1^−^* 2636 virus (Figure 1B) resulted in clonal LCLs, which proliferated in the absence of either CD40L or IL-4 signals, indicating that preactivated B cells compensate for the functional defect of a *vBcl-2^−^* virus (Figure 1E). Once established, these LCLs infected with *vBcl2^−^* EBV proliferated normally and did not show an apparently different phenotype when compared with wild-type EBV-infected LCLs (data not shown).

### *vBcl-2* Genes Are Maximally Expressed Very Early After EBV Infection of Primary B Cells

Eleven EBV genes have previously been identified as being constitutively expressed in established LCLs. Neither *BALF1* nor *BHRF1* is among this group, however [15,36]. Latent EBV genes such as *EBNA2* and *LMP1* encode key regulators of cellular proliferation, which are required to initiate and maintain proliferation of lymphoblastoid cells in vitro [22,24–26,37]. Therefore, we set out to analyze the expression kinetics of *BALF1* and *BHRF1* in infected primary B cells. As shown in Figure 2, upon infection with the B95.8 prototype EBV strain, *BHRF1* and *BALF1* transcripts were readily detected within 24 h postinfection (p.i.) but not in cells infected at 4 °C or in uninfected cells (Figure 2A and 2E). Whereas *EBNA2* is constitutively expressed in established latently infected LCLs (data not shown [15]), the expression of both *BALF1* and *BHRF1* rapidly decreased, and their transcripts were weakly or not detected by RT-PCR 3 wk p.i. (Figure 2A and 2E). *EBNA2* transcripts could be detected 1 d p.i. in wild-type 2089 EBV or 2636 *BALF1^−^*/*BHRF1^−^* mutant infected primary B cells (Figure 2B). Similarly, *vBcl-2* transcripts are readily detectable in cells infected with an *EBNA2^–^* 2491 mutant EBV (Figure 2C), indicating that *EBNA2* and *vBcl-2* genes are independently expressed very early after infection.

**Figure 2:**
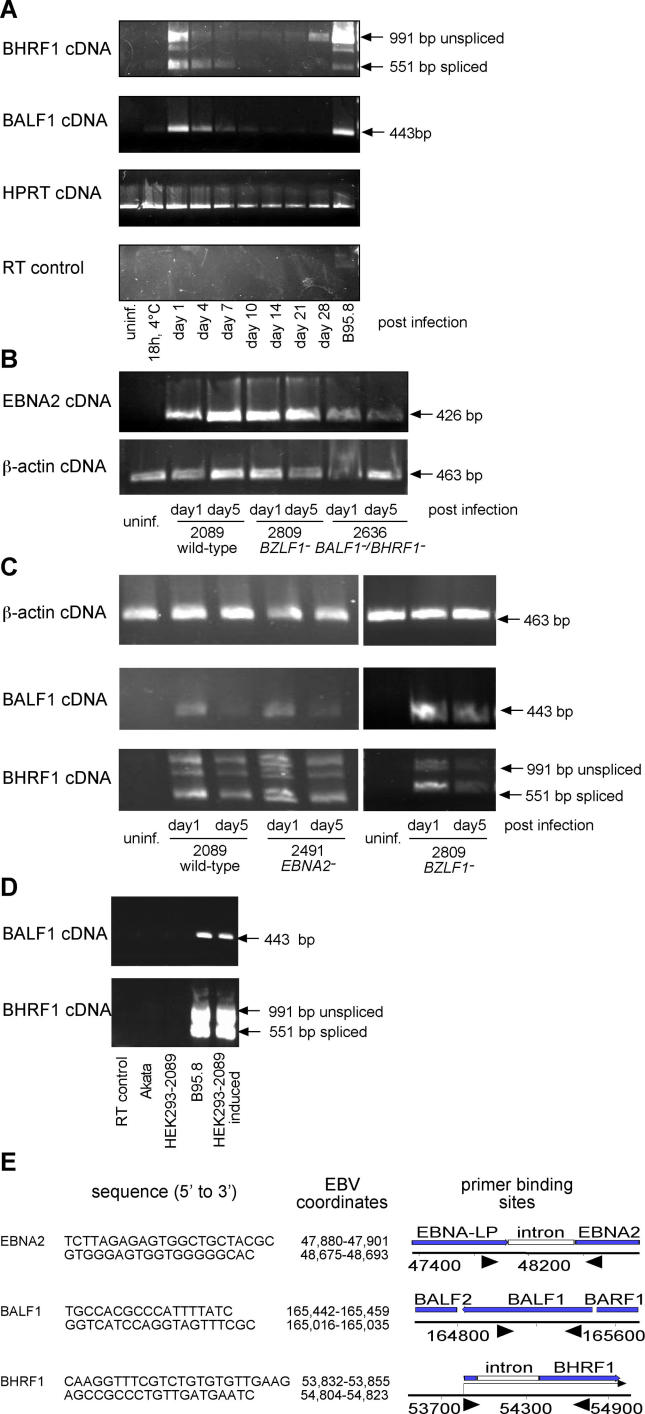
Detection of EBNA2, BALF1, and BHRF1 Transcripts by RT-PCR Analysis (A). Primary B cells were infected with B95.8 EBV, and RNA was prepared at various time points. The expression of BALF1, BHRF1, and HPRT (as control) was analyzed by RT-PCR. (B). Primary B cells were infected with wild-type 2089 EBV, the *BZFL1^−^* mutant 2809, or the *BALF1^−^*/*BHRF1^−^* mutant 2636 (MOI 0.1), and the expression of EBNA2 was determined by RT-PCR. EBNA2 mRNA was detectable in comparable amounts in cells infected with either virus stock on day 1 and on day 5 p.i. (C). Primary B cells were infected with wild-type 2089 EBV, the *BZFL1^−^* mutant 2809, or the *EBNA2^−^* mutant 2491. Similar to (A), vBcl-2 transcripts were detectable in comparable amounts in cells infected with either virus stock on day 1 and on day 5 p.i. but at a reduced level on day 5. (D). BALF1 and BHRF1 transcript levels in latently infected and lytically induced cell lines. Neither BALF1 nor BHRF1 are detectably expressed in the latently infected, EBV-positive Akata cell line [62] or in 293HEK cells stably transfected with wild-type 2089 EBV. Both mRNAs are easily detectable in B95.8 cells, which spontaneously support productive infection, or in lytically induced 293HEK cells carrying the maxi-EBV 2089). (E) Schematic overview of the PCR primers and their location used to detect EBNA2-, BALF1-, or BHRF1-specific cDNAs. The PCR product indicative of correctly spliced EBNA2 transcripts is 426 bp in length; the BALF1 PCR product is 443 bp in length. BHRF1-specific PCR products are expected to be 551 bp and 991 bp in length, representing the spliced and unspliced transcripts, respectively.

As expected, *BALF1* and *BHRF1* were not expressed in other latently EBV-infected cells (Figure 2D) but readily detectable in cells that spontaneously support the lytic phase of EBV's life cycle (B95.8 cells in Figure 2A and 2D) and maximally expressed in cells in which the lytic phase was induced (Figure 2D). Because both *vBcl-2* genes were induced during EBV's lytic phase, and several reports indicate that lytic expression of *BHRF1* is strictly dependent on the immediate-early transcriptional activator BZFL1 ([15,38] and references therein), we wanted to learn whether initial expression of *BALF1* or *BHRF1* in newly infected primary B cells is also regulated by *BZLF1. BZLF1* encodes a molecular switch protein, which is instrumental in inducing the lytic phase in latently EBV-infected cells ([15] and references therein). The EBV mutant 2809, which is *BZLF1^−^* [39], was used to infect primary B cells, and the expression of *BALF1* and *BHRF1* was assessed by RT-PCR. Again, *vBcl-2* transcripts were readily detectable in cells infected with the *BZFL1^−^* 2809 EBV mutant, indicating that BZLF1 does not regulate *BALF1* and *BHRF1* expression (Figure 2C). As expected, *EBNA2* expression was also *BZLF1* independent (Figure 2B).

### Cell Cycle Activation Is Abrogated in *vBcl-2^–^* EBV-Infected Cells

We questioned whether *vBcl-2* gene products might be critical for cell cycle entry similar to EBNA2 [15,40]. Stocks of the *BALF1^−^*/*BHRF1^−^* 2636 virus were used to infect primary B cells at a multiplicity of infection (MOI) of 0.5 GRU with the *EBNA2^−^* mutant 2491 or wild-type EBV 2089 serving as controls. For direct comparison, a fraction of the cells was left uninfected. The cell cycle status of the cells was analyzed by 5-bromo-2′-deoxyuridine (BrdU)-incorporation and fluorescence-activated cell-sorting (FACS) analysis, which revealed cycling cells in wild-type EBV-infected cells as early as day 3 p.i. (Figure 3). Uninfected cells did not enter the cell cycle nor did the cells infected with the *EBNA2^−^* or the *BALF1^−^*/*BHRF1^−^* 2636 mutants. These latter infected cells all showed a rapid increase in the fraction of cells with a subG_1_ DNA content indicating that the inactivation of both *vBcl-2* genes prevented cell cycle entry similar to *EBNA2^−^* EBV, which lacks the key regulator of EBV's latent genes (Figure 3).

**Figure 3:**
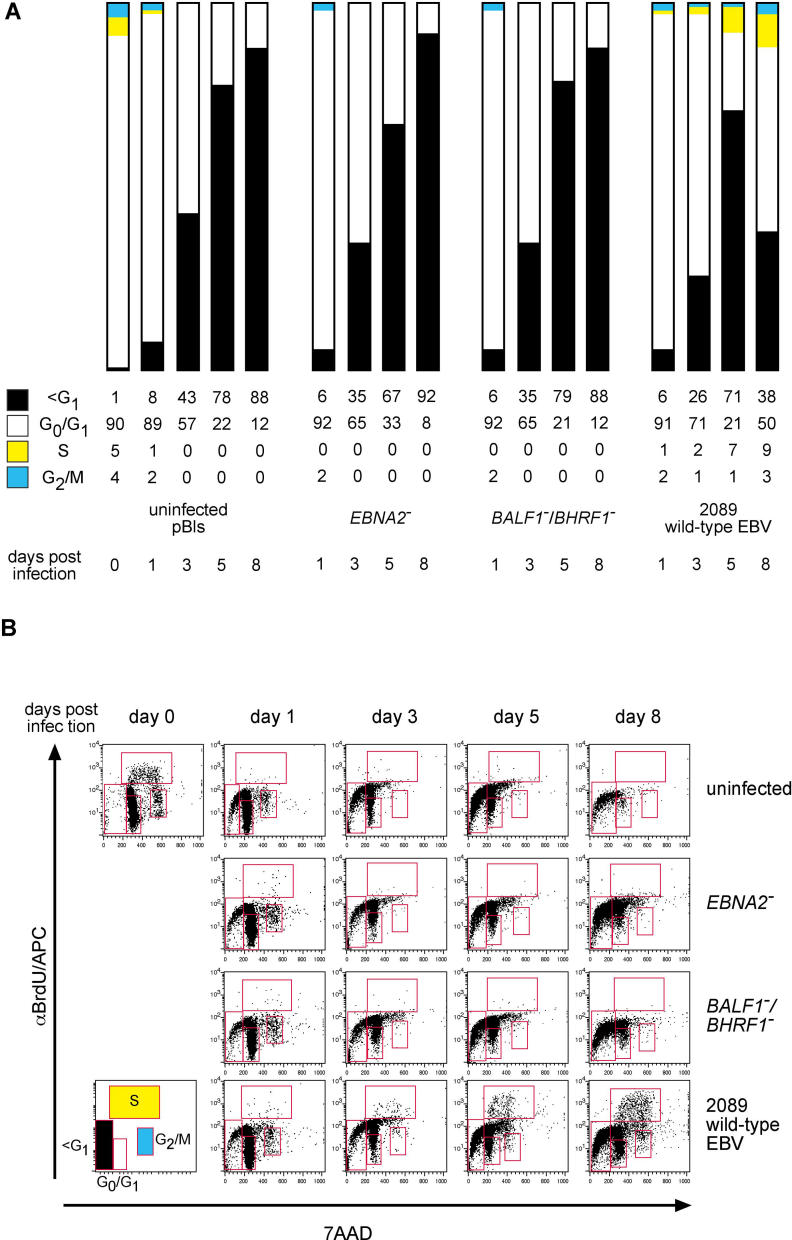
Cell Cycle Analysis of Uninfected Primary B Cells (PBls) or PBls Infected with *EBNA2^−^* Virus, *BALF1^−^*/*BHRF1^−^* Mutant 2636, or Wild-Type 2089 EBV Primary B cells (10^5^) per time point were infected with a viral dose such that about 50% of the cells were infected or left uninfected, and their cell cycle profiles were determined by BrdU incorporation and FACS analysis. Only wild-type 2089 EBV induced cell cycle progression, whereas uninfected cells as well as cells infected with the two mutants became apoptotic as indicated by their subG_1_ DNA content. (A) shows the compiled data of the primary results, which are illustrated in (B). One representative experiment out of three is shown.

### B-Cell Apoptosis Is Delayed in *vBcl-2^+^* EBV-Infected Primary B Cells

Given that EBNA2 is a pivotal mediator of EBV's known latent genes, it is important to know if EBV's *vBcl-2* genes, which are required to establish latency (see Figure 1), depend on any of EBNA2′s functions. We tested whether primary B cells infected with the *EBNA2^–^* mutant, which is capable of expressing both *vBcl-2* genes (see Figure 2C), showed a phenotype different from cells infected with a mutant null for *vBcl-2*s. Because both viral mutants are deficient in inducing cellular proliferation (see Figure 3), we could concentrate on effects related to apoptotic markers independent of global cellular activation (Figure 4). Toward this end, we infected primary B cells with the *EBNA2^−^* 2491 or the *BALF1^−^*/*BHRF1^−^* 2636 viruses (or wild-type 2089 EBV as a positive control) and determined the occurrence of GFP^+^ EBV-infected cells by FACS (Figure 4C). To monitor the numbers of GFP^+^ cells over a period of 8 d, calibration beads were used as an internal reference (Figure 4A). By three-parameter FACS analysis, GFP^+^ B cells were also analyzed for binding of Annexin-V and propidium iodine (PI) uptake as indicators of early apoptosis and loss of membrane integrity, respectively (Figure 4D). In parallel, uninfected cells with typical lymphocytic characteristics by forward and sideward scatter criteria (Figure 4B) were compared for their Annexin-V and PI staining. As shown in Figure 4D and 4E, uninfected primary B cells as well as those infected with *BALF1^−^*/*BHRF1^−^* 2636 mutant rapidly lost their viability. As early as day 3 p.i., about 70% of the *BALF1^−^*/*BHRF1^−^* virus-infected cells were Annexin-V^+^/PI^+^ comparable to uninfected cells. By day 5, few lymphocytes had survived and none were found on day 8 (Figure 4E). In stark contrast, all primary B cells infected with the *EBNA2^−^* mutant 2491, which expressed *BALF1* and *BHRF1* transcripts immediately after infection (see Figure 2C), were alive on day 3 p.i., about 15% survived until day 5, and a few percent of the cells were GFP^+^/Annexin-V^−^/PI^−^ 8 d p.i. (Figure 4D and 4E). Thus, EBV's *Bcl-2* homologs support initial B-cell survival and rescue EBV-infected B cells, which would otherwise succumb rapidly to spontaneous apoptosis.

**Figure 4:**
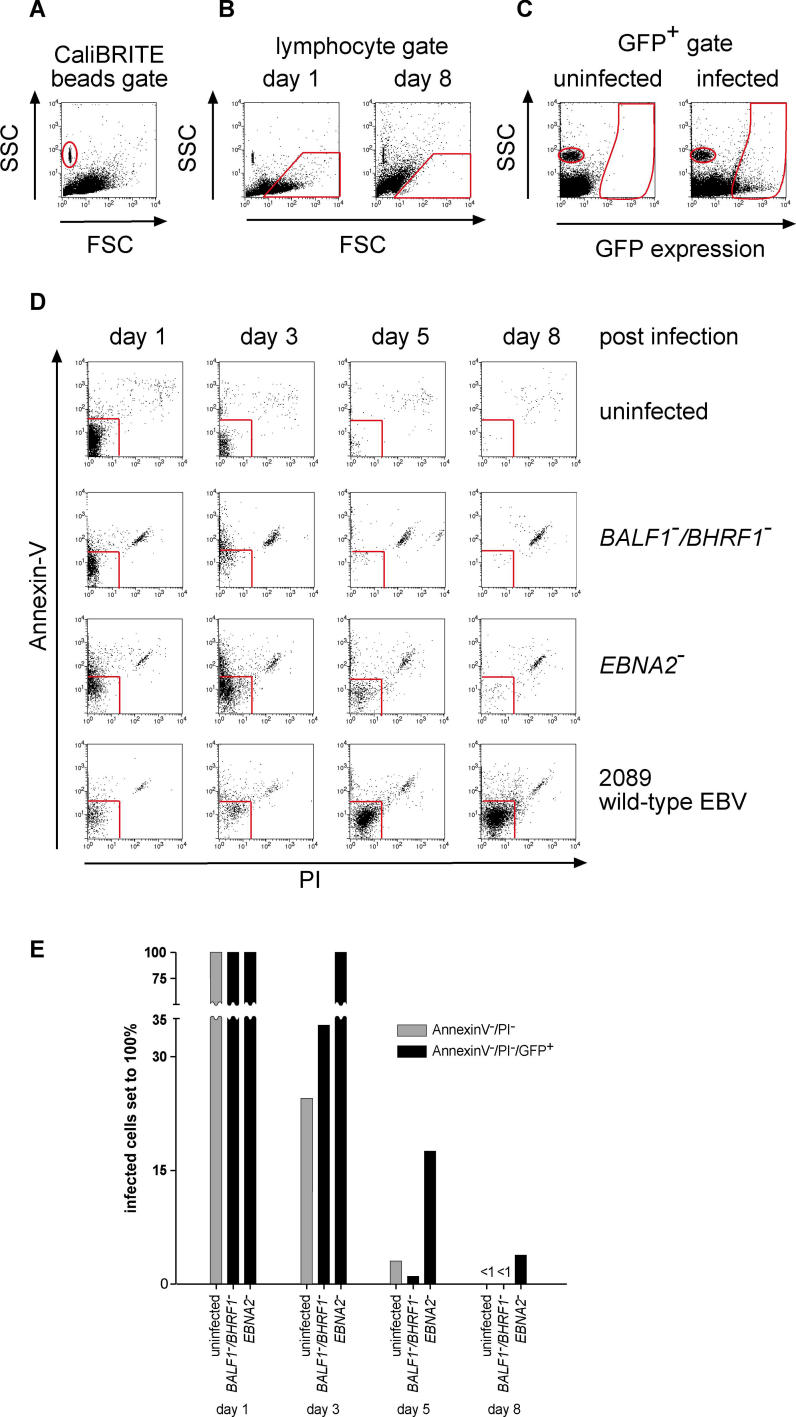
Apoptosis of Primary B Cells Primary B cells were infected with the *EBNA2^−^* mutant 2491, the *BALF1^−^*/*BHRF1^−^* mutant 2636, or wild-type 2089 EBV, at an MOI of 0.1, or the cells were left uninfected. Cells were analyzed by FACS at days 1, 3, 5, and 8 p.i. (A) Total cellular events were collected until 3 × 10^3^ BD CaliBRITE beads as a volume standard were counted. These beads, which are indicated by red circles in the FACS diagrams in (A) and (C), were used as an internal volume reference corresponding to 3 × 10^4^ cells plated initially. (B) Uninfected cells that fulfilled the criteria of lymphocytes according to their forward (FSC) and sideward (SSC) scatter characteristics were gated as indicated. One day after B-cell preparation, lymphocytes were present in the lymphocyte gate as expected, but only a few lymphocytes were still present in this gate 8 d after cell preparation when the cells had been left uninfected. Similarly, EBV-infected lymphocytes were selected according to the same scatter criteria (data not shown). (C) EBV-infected GFP^+^ cells were gated as indicated. The example shows an uninfected and a 2491 *EBNA2^−^* EBV-infected B-cell sample with 3 × 10^3^ BD CaliBRITE beads added 1 d p.i. (D) Uninfected primary B cells within the lymphocyte gate or EBV-infected GFP^+^ lymphocytes were analyzed by FACS for Annexin-V binding and PI staining at different time points p.i. The absolute numbers of Annexin-V^−^/PI^−^/GFP^+^ cells allowed the calculation of surviving cells at each time point p.i. Uninfected cells indicate the rate and kinetics of spontaneous apoptosis of primary B cells ex vivo. Primary B cells infected with the *BALF1^−^*/*BHRF1^−^* mutant 2636 died as rapidly as uninfected cells. Only B cells infected with the *EBNA2^−^* mutant 2491, which is *vBcl-2^+^,* survived considerably longer. Primary B cells infected with wild-type 2089 EBV as a positive control rapidly increased in the number of Annexin-V^−^/PI^−^/GFP^+^ cells. (E) Summary of the primary data shown in (D). Annexin-V^−^/PI^−^ cells (uninfected control to determine spontaneous programmed cell death), or Annexin-V^−^/PI^−^/GFP^+^ (infected) cells were set to 100% at day 1, and the percentile of Annexin-V^−^/PI^−^ cells at each time point was calculated. One representative experiment out of three is shown.

## Discussion

### *vBcl-2* Genes Are Essential for Growth Transformation of Primary B Lymphocytes

Some viruses encode proteins that interfere with the host's apoptotic machinery to ensure short-term cell survival and to yield progeny before the cell lyses and dies [3,41,42]. Similarly, γ-herpesviruses encode *vBcl-2* genes, whose gene products are antiapoptotic Bcl-2 family members and act during productive infection or contribute to viral virulence [10,12]. Only EBV is known to encode two *Bcl-2* homologs, *BALF1* and *BHRF1. BHRF1* was shown to be dispensable for B-cell growth transformation [43,44], but its product does have antiapoptotic functions [4,45]. The function of the *BALF1* gene product is controversial [5,6]. Our experiments indicate that (i) both *BALF1* and *BHRF1* belong to the same genetic complementation group and are functionally redundant (see Figure 1). Either *BALF1* or *BHRF1* is (ii) essential to establish latently infected LCLs (see Figures 1 and 3) but (iii) dispensable for their continued proliferation (see Figure 1E) and (iv) generation of virus progeny (data not shown). In newly infected primary B cells, (v) both *vBcl-2* genes are expressed immediately but transiently after infection (see Figure 2), in contrast to the group of 11 latent EBV genes, which are constitutively expressed [15,46]. Surprisingly, BALF1 and BHRF1 proteins (vi) prevent spontaneous programmed cell death in newly infected resting primary B cells (see Figure 4). This newly identified function of vBcl-2 ensures successful infection of primary B cells, gives rise to latent infection, and supports growth transformation of resting B cells in vitro.

This finding seems to be without precedent because vBcl-2 homologs have not been recognized to contribute to viral latent infection directly [2,8,9], probably because herpesviral latency can only be studied with a few viruses. Similar to EBV, long-term latency of the murine γ-herpesvirus MHV-68 is narrowly confined to cells expressing a B-cell phenotype [47,48]. Infection of mice with MHV-68 provides a tractable and authentic animal model to study herpesviral latency in the context of its natural host. A viral mutant deficient for the MHV-68 *vBcl-2* gene M11 was found compromised in reactivating from latency [12], whereas a more recent paper identified a *vBcl-2* associated deficit in establishment of latency [10]. Although the molecular basis for these observations remains unclear, both seem to be consistent with vBcl-2 protecting latently infected B cells from apoptotic death. Similar to our findings, no role for *vBcl-2* of MHV-68 was apparent during acute viral replication in vivo or viral virulence [10–12].

### How Are *vBcl-2* Genes Regulated in Newly Infected Primary B Cells?

Experimental data clearly demonstrate that *BALF1* and *BHRF1* are maximally expressed in primary B cells within 24 h p.i. (see Figure 2A). Because the expression of *BALF1* and *BHRF1* is also prominent during EBV's lytic phase (see Figure 2D), we wondered whether their expression might be regulated by the viral immediate-early gene *BZLF1.* BZLF1 is a transcription factor and acts like a molecular switch to induce the lytic phase in latently EBV-infected cells ([15] and references therein). Unexpectedly, the expression of both *vBcl-2* genes was found to be independent of *BZLF1* (see Figure 2C). Thus, we do not know the mechanisms regulating the expression of both *BALF1* and *BHRF1,* but we would like to speculate that both genes are directly and spontaneously expressed from the transducing EBV genome. The herpesviral DNA delivered to the nucleus of the infected cell is unmethylated, coated with polyamines [49], and not in a chromatin-like state [50], which should permit universal access of the transcriptional machinery to many viral promoter elements. Modifications of the EBV genomic DNA over time will alter its accessibility, which might cause a rapid decline in the expression of *BALF1* and *BHRF1* when the virus eventually establishes a genuine latent infection (see Figure 2A).

### vBcl-2 Homologs Are Likely to Be Involved in In Vivo Latency and Oncogenesis of EBV-Associated Tumors

EBV-associated B-cell lymphomas originate from germinal center B cells, which are inherently prone to apoptosis as they undergo affinity maturation and somatic hypermutation in their B-cell receptor genes, events characteristic of this stage of B-cell differentiation. It is unclear whether EBV infects naïve B cells prior to their germinal center passage [51,52] or germinal center B cells directly [53]. Our data indicate in either case that BALF1 and BHRF1 are involved in the in vivo survival of EBV-infected CD77^+^ germinal center B cells. These cells normally are highly sensitized to apoptotic signals, and most will be eliminated via programmed cell death [54]. EBV is expected to provide an important signal or signals to prevent apoptosis common to germinal center B cells because a fraction of EBV-infected and malignant tumor cells contain only nonfunctional B-cell receptor genes and would have been expected to undergo programmed cell death. EBV's vBcl-2 proteins likely constitute this survival signal, which would be superseded by the eventual expression of LMP2A [55,56] and/or other “classical” latent EBV genes. Thus, EBV's Bcl-2 homologs are likely to counteract the physiological elimination of B cells, which might be a crucial viral contribution to lymphomagenesis [13].

The physiological expansion of B cells during the germinal center reaction increases the size of the pool of EBV-infected cells, which go on to differentiate into long-lived memory B cells, the reservoir for virus in vivo [51,52]. Because memory B cells originate from functional survivors of the germinal center passage, we favor the notion that during this passage EBV's antiapoptotic viral genes provide a survival advantage to the infected cells favoring their developing into memory B cells in which EBV is latent and can thereby establish a lifetime relationship with its human host.

## Materials and Methods

### Construction of viral mutants

The *BALF1* ^−^ and *BHRF1^−^* single deletion mutants were constructed on the basis of the wild-type B95.8 EBV strain cloned onto the F-factor plasmid p2089 [32]. The inactivation of the *vBcl-2* genes or the *EBNA2* gene in the EBV genome was carried out with two different genetic procedures as described in a recent review [57]. To promote the integration of linear DNA fragments in the DH10B *Escherichia coli* host, the conditional *recA/redγ* expression plasmid p2650 based on the pST76-amp plasmid was employed. To generate the *vBcl-2* targeting plasmids for homologous recombination, subgenomic EBV DNA fragments of about 10 kilobasepairs (kbp) in size, spanning either the *BALF1* or the *BHRF1* locus, were subcloned to yield the plasmids p1352 and p528, respectively. The *BALF1* targeting plasmid p2564 was generated by inserting the tetracycline resistance gene from pCP16 [58] into the *BALF1* open reading frame in p1352. The kanamycin phosphotransferase gene from pCP15 [58] was inserted into the *BHRF1* coding sequence of p528, giving rise to the targeting plasmid p2632. Homologous recombinations were carried out with the p2089 maxi-EBV genome to yield the EBV mutant genomes p2512 and p2637, which carry singly inactivated *BALF1* and *BHRF1* genes, respectively (see Figure 1A and 1B). The two *BALF1^−^*/*BHRF1^−^* and *BHRF1^−^*/*BALF1^−^* mutants p2636 and p2765 were generated in a second round of homologous recombination with the two targeting plasmids p2632 and p2564 (see Figure 1A and 1B). The *BALF1^+^*/*BHRF1^−^* revertant maxi-EBV p3227 with a reconstructed wild-type *BALF1* gene was generated with the aid of the pST76-amp-based shuttle plasmid [59] carrying the wild-type *BALF1* gene flanked by homologous EBV sequences. A cointegrate of the shuttle plasmid and the p2636 EBV plasmid was formed with the “chromosomal-building” technique [60], which led to the maxi-EBV construct p3227 after routine genetic manipulations. The *EBNA2^−^* EBV mutant 2491 was also constructed with the chromosomal-building technique and the shuttle plasmid p2419.1 to delete the entire coding sequences of the *EBNA2* gene in the context of the maxi-EBV genome 2089. The *BZLF1^–^* EBV mutant 2809 was described [39]. The genetic compositions of the modified EBV genomes were verified by restriction enzyme analysis, Southern blot hybridization, and partial DNA sequencing. Details for the generation of all EBV mutants are available upon request.

### Preparation and quantification of infectious viral vector stocks

On the basis of 293HEK cells, stable cell lines were established after individual transfection of the genomic maxi-EBV DNAs and subsequent selection with hygromycin as described [29]. To obtain virus stocks, the cell lines were transiently transfected with expression plasmids encoding *BZLF1* [61] and *BALF4* [31] to induce EBV's lytic cycle. Four days p.i. supernatants were harvested and filtered through 1.2-μm pore filters. The different EBV vector stocks were quantified by infection of Raji cells as described [29,31]. Briefly, 3 × 10^5^ Raji cells were incubated at 37 °C in 24-well cluster plates with different dilutions of the virus stocks to be analyzed. The absolute number of GFP^+^ cells was determined by ultraviolet microscopy in a defined fraction of the total Raji cell population 4 d after infection. On the basis of these data, GRUs per milliliter were calculated as a measure of the concentration of infectious maxi-EBV particles in different virus stocks. We used this titering method because other biologically relevant readouts are not feasible with nontransforming EBV mutants and very time-consuming with transformation-competent wild-type EBV. Infection and evaluation of GFP^+^ Raji cells underestimates the concentration of infectious EBV virions by a factor of at least ten (data not shown).

### Isolation, separation, and infection of primary B lymphocytes

Human primary mononuclear cells were isolated from adenoids, depleted of T cells by rosetting with sheep erythrocytes and purified by Ficoll-Hypaque density-gradient centrifugation. The cell population was further depleted of monocytes by plastic adherence for 1 h. To quantify the efficiency of growth transformation with the different virus stocks, 3 × 10^5^ human primary B lymphocytes per well were seeded in 96-well cluster plates on lethally irradiated Wi38 human fibroblast feeder cells and infected with serially diluted virus stocks with 2089 wild-type EBV and the different viral mutants in a total volume of 100 μl as described [29]. Forty-eight wells were infected with each single virus dilution. A 50-μl portion of medium was exchanged every week for fresh medium, and the number of wells with proliferating cells was determined 6 wk p.i. The infection experiments with different viral mutants were independently performed at least in triplicate. The experiments with activated B blasts were carried out similarly. In order to generate proliferating and activated B blasts, primary B lymphocytes were cultivated on an irradiated CD40-ligand feeder cell layer [34] in the presence of 4 ng/ml IL-4 (PAN Biotech, Aidenbach, Germany) and 1 μg/ml cyclosporine A (Novartis, Basel, Switzerland) for several weeks prior to the infection experiments. The proliferation of B blasts and their long-term survival in vitro is strictly dependent on both CD40L and IL-4 generated signals. To quantify the number of GRUs needed to yield clonal LCLs from B blasts, 3 × 10^5^ blasts per well were seeded on an irradiated CD40-feeder layer in the absence of IL-4 and infected with serially diluted virus stocks as described above. Fifty percent of the volume of the cell culture media was exchanged on a weekly basis, and the cells were cultivated for 6 wk, during which the CD40-feeder cell layer had completely disintegrated.

### RT-PCR analysis

RNA was extracted from primary B cells with the RNeasy Midi Kit (Qiagen, Valencia, California, United States); 5 μg of RNA was reverse transcribed with the Superscript III First Strand Synthesis Kit (Invitrogen, Carlsbad, California, United States) according to the manufacturer's protocol in a total volume of 20 μl. To monitor cellular DNA contamination of the RNA preparation, PCR reactions were performed with primers for the abundant cellular transcripts HPRT and ß-actin. PCR reactions for ß-actin were an initial template denaturation of 4 min at 94 °C, with amplification for 25 cycles (1 min at 94 °C, 1 min at 61 °C, 1 min at 72 °C), followed by a final elongation for 10 min at 72 °C. PCR reactions for HPRT were 4 min at 94 °C, with amplification for 25 cycles (1 min at 94 °C, 1 min at 54 °C, 1 min at 72 °C), followed by a final elongation for 10 min at 72 °C. Two out of 20 μl of the cDNA reaction were used as template. Unspliced PCR amplification products indicative of cellular DNA contamination were not detected (see Figure 2). PCR reactions for cDNA detection of three EBV genes were as follows: *EBNA2* (an initial template denaturation of 4 min at 94 °C; 35 cycles of 1 min at 94 °C, 1 min at 61 °C, 1 min at 72 °C; followed by a final elongation for 10 min at 72 °C); *BHRF1* (an initial template denaturation of 4 min at 94 °C; 35 cycles of 1 min at 94 °C, 1 min at 54 °C, 1.5 min at 72 °C; followed by a final elongation for10 min at 72 °C); and *BALF1* (an initial template denaturation of 4 min at 94 °C; 35 cycles of 1 min at 94 °C, 1 min at 61 °C, 1 min at 72 °C; followed by a final elongation for 10 min at 72 °C). Oligonucleotide primer sequences for the EBV genes *EBNA2, BALF1,* and *BHRF1* are provided in Figure 2E together with their nucleotide coordinates of the EBV strain B95.8. The primer sequences for HPRT cDNA detection were 5′- GAGCTATTGTAATGACCAGTC-3′ and −5′- CCAAACTCAACTTGAACTCTC-3′; ß-actin cDNA was amplified with the primers 5′- CACCCTGTGCTGCTCACCGAGGCC-3′ and 5′- ACCGCTCGTTGCCAATAGTGATGA-3′.

### Cell cycle analysis and analysis of apoptosis

Primary B cells (4 × 10^5^) were infected with 4 × 10^4^ GRUs of the different virus stocks in 20 ml of culture medium in order to obtain an MOI of 0.1. The cells were kept on plastic, and at days 1, 3, 5, and 8 one-fourth of the culture (5 ml) was harvested and further analyzed for cell cycle status and apoptosis. Infection experiments were carried out with 2089 EBV, the *EBNA2^−^* deletion mutant 2491, and the different mutant virus stocks (see Figure 1); for a negative control the cells were left uninfected. To determine the fraction of apoptotic cells, Annexin-V staining was performed with the Annexin-V-APC kit (BioVision, Mountain View, California, United States), according to the manufacturer's protocol. For each time point, 5 ml of each sample was harvested, spun, and the cells were stained with 5 μl of APC-coupled Annexin-V and 5 μl of PI at a final concentration of 10 μg/ml. As an internal FACS calibration standard, 1 × 10^4^ BD CaliBRITE Beads (Becton-Dickinson, Palo Alto, California, United States) were added to yield a final concentration of 2 × 10^4^ beads/ml. The beads are very small, resulting in a high intensity in the sideward scatter channel. Since the beads also display a very bright APC fluorescence, their characteristics do not interfere with the cells to be analyzed but allow their unbiased detection and quantification. FACS analysis was carried out in a FACS-Calibur machine (Becton-Dickinson). The 3 × 10^3^ BD CaliBRITE Beads were set as a volume standard corresponding to 3 × 10^4^ cells plated initially to determine the absolute number of cells at any given time point without interference from the dynamics of the cell culture. To exclude cellular debris, only cells within the lymphocyte gate were analyzed (see Figure 4B).

Cells infected with the different recombinant virus stocks express GFP as early as day 1 p.i. The GFP gate was set such that only cells with a GFP signal brighter than primary B cells infected with the GFP^−^ prototypic B95.8 EBV strain scored positive as infected GFP^+^ cells (see Figure 4C). EBV-infected GFP^+^ cells in the lymphocyte gate were analyzed for their Annexin-V and PI staining. In uninfected samples, only cells that fulfilled the criteria of lymphocytic cells by forward and sideward scatter criteria were analyzed for both Annexin-V and PI staining. The number of Annexin-V^−^/PI^−^ cells in the infected samples and the uninfected control were set to 100% at day 1 p.i.

To analyze the cell cycle status of the infected primary B cells in comparison to the uninfected controls, the samples were incubated with the thymidine analog BrdU for 2 h prior to FACS analysis at each time point. The cell proliferation assays were immediately performed with the BrdU Flow Kit (BD Biosciences Pharmingen, San Diego, California, United States). The cells were stained with an APC-coupled BrdU-specific antibody after fixation and permeabilization, and the cellular DNA was counterstained with the DNA intercalating dye 7-AAD according to the manufacturer's protocol. FACS analysis was performed until 3 × 10^4^ cells were analyzed. The recorded data were gated for cells in the G1, S, G2/M phases of the cell cycle and for cells with a subG1 DNA content. The total of all events was set to 100%.

## Supporting Information

### Accession Numbers

UniProtKB/TrEMBL (http://www.expasy.org/uniprot/) accession numbers for the proteins are Bax (Q07812), Bcl-X_L_ (Q07817), Bcl-2 (P10415), CD40 receptor (P25942), LMP1 (P03230), LMP2A (Q777H4), and LMP2B (Q8AZK9); for the virus, E1B 19K (P03247); for the genes, *BALF1* (Q777A6) and *BHRF1* (Q777H0); for EBV nuclear antigens, EBNA1 (P03211), EBNA2 (Q69023), EBNA3A (Q8AZJ8), EBNA3B (Q777E8), EBNA3C (Q777E7), and EBNA-LP (Q8AZK7).

## Abbreviations

BrdU: 5-bromo-2′-deoxyuridine
EBV: Epstein-Barr virus
FACS: fluorescence-activated cell sorting
GFP: green fluorescence protein
GRU: green Raji unit
kbp: kilobasepairs
LCL: lymphoblastoid cell line
MOI: multiplicity of infection
p.i.: postinfection
PI: propidium iodine
vBcl-2: viral Bcl-2

## References

[pbio-0030404-b01] Cuconati A, White E (2002). Viral homologs of BCL-2: Role of apoptosis in the regulation of virus infection. Genes Dev.

[pbio-0030404-b02] Boya P, Pauleau AL, Poncet D, Gonzalez-Polo RA, Zamzami N (2004). Viral proteins targeting mitochondria: Controlling cell death. Biochim Biophys Acta.

[pbio-0030404-b03] Benedict CA, Norris PS, Ware CF (2002). To kill or be killed: Viral evasion of apoptosis. Nat Immunol.

[pbio-0030404-b04] Henderson S, Huen D, Rowe M, Dawson C, Johnson G (1993). Epstein-Barr virus-coded BHRF1 protein: A viral homologue of Bcl-2, protects human B cells from programmed cell death. Proc Natl Acad Sci U S A.

[pbio-0030404-b05] Bellows DS, Howell M, Pearson C, Hazlewood SA, Hardwick JM (2002). Epstein-Barr virus BALF1 is a BCL-2-like antagonist of the herpesvirus antiapoptotic BCL-2 proteins. J Virol.

[pbio-0030404-b06] Marshall WL, Yim C, Gustafson E, Graf T, Sage DR (1999). Epstein-Barr virus encodes a novel homolog of the bcl-2 oncogene that inhibits apoptosis and associates with Bax and Bak. J Virol.

[pbio-0030404-b07] Irusta PM, Chen YB, Hardwick JM (2003). Viral modulators of cell death provide new links to old pathways. Curr Opin Cell Biol.

[pbio-0030404-b08] Hilleman MR (2004). Strategies and mechanisms for host and pathogen survival in acute and persistent viral infections. Proc Natl Acad Sci U S A.

[pbio-0030404-b09] Roulston A, Marcellus RC, Branton PE (1999). Viruses and apoptosis. Annu Rev Microbiol.

[pbio-0030404-b10] de Lima BD, May JS, Marques S, Simas JP, Stevenson PG (2005). Murine gammaherpesvirus 68 bcl-2 homologue contributes to latency establishment in vivo. J Gen Virol.

[pbio-0030404-b11] Gangappa S, Kapadia SB, Speck SH, Virgin HW (2002). Antibody to a lytic cycle viral protein decreases gammaherpesvirus latency in B-cell-deficient mice. J Virol.

[pbio-0030404-b12] Gangappa S, van Dyk LF, Jewett TJ, Speck SH, Virgin HW (2002). Identification of the in vivo role of a viral bcl-2. J Exp Med.

[pbio-0030404-b13] Küppers R (2003). B cells under influence: Transformation of B cells by Epstein-Barr virus. Nat Rev Immunol.

[pbio-0030404-b14] Babcock GJ, Hochberg D, Thorley-Lawson AD (2000). The expression pattern of Epstein-Barr virus latent genes in vivo is dependent upon the differentiation stage of the infected B cell. Immunity.

[pbio-0030404-b15] Kieff E, Rickinson AB, Knipe DM, Howley PM, Griffin DE, Martin MA, Lamb RA (2001). Epstein-Barr virus and its replication. Fields' virology, 4th ed.

[pbio-0030404-b16] Rickinson AB, Kieff E, Knipe DM, Howley PM, Griffin DE, Martin MA, Lamb RA (2001). Epstein-Barr virus. Fields' virology, 4th ed.

[pbio-0030404-b17] [Anonymous] (1997). Epstein-Barr virus and Kaposi's sarcoma herpesvirus/human herpesvirus 8; International Agency for Research on Cancer.

[pbio-0030404-b18] Dirmeier U, Hoffmann R, Kilger E, Schultheiss U, Briseno C (2005). Latent membrane protein 1 of Epstein-Barr virus coordinately regulates proliferation with control of apoptosis. Oncogene.

[pbio-0030404-b19] Cahir-McFarland ED, Davidson DM, Schauer SL, Duong J, Kieff E (2000). NF-kappa B inhibition causes spontaneous apoptosis in Epstein-Barr virus-transformed lymphoblastoid cells. Proc Natl Acad Sci U S A.

[pbio-0030404-b20] Cahir-McFarland ED, Carter K, Rosenwald A, Giltnane JM, Henrickson SE (2004). Role of NF-kappa B in cell survival and transcription of latent membrane protein 1-expressing or Epstein-Barr virus latency III-infected cells. J Virol.

[pbio-0030404-b21] Feuillard J, Schuhmacher M, Kohanna S, Asso-Bonnet M, Ledeur F (2000). Inducible loss of NF-kappaB activity is associated with apoptosis and Bcl-2 down-regulation in Epstein-Barr virus-transformed B lymphocytes. Blood.

[pbio-0030404-b22] Kilger E, Kieser A, Baumann M, Hammerschmidt W (1998). Epstein-Barr virus-mediated B-cell proliferation is dependent upon latent membrane protein 1, which simulates an activated CD40 receptor. EMBO J.

[pbio-0030404-b23] Kulwichit W, Edwards RH, Davenport EM, Baskar JF, Godfrey V (1998). Expression of the Epstein-Barr virus latent membrane protein 1 induces B cell lymphoma in transgenic mice. Proc Natl Acad Sci U S A.

[pbio-0030404-b24] Hammerschmidt W, Sugden B (1989). Genetic analysis of immortalizing functions of Epstein-Barr virus in human B lymphocytes. Nature.

[pbio-0030404-b25] Cohen JI, Wang F, Mannick J, Kieff E (1989). Epstein-Barr virus nuclear protein 2 is a key determinant of lymphocyte transformation. Proc Natl Acad Sci U S A.

[pbio-0030404-b26] Kaye KM, Izumi KM, Kieff E (1993). Epstein-Barr virus latent membrane protein 1 is essential for B-lymphocyte growth transformation. Proc Natl Acad Sci U S A.

[pbio-0030404-b27] Tomkinson B, Robertson E, Kieff E (1993). Epstein-Barr virus nuclear proteins EBNA-3A and EBNA-3C are essential for B-lymphocyte growth transformation. J Virol.

[pbio-0030404-b28] Kempkes B, Pich D, Zeidler R, Sugden B, Hammerschmidt W (1995). Immortalization of human B lymphocytes by a plasmid containing 71 kilobase pairs of Epstein-Barr virus DNA. J Virol.

[pbio-0030404-b29] Dirmeier U, Neuhierl B, Kilger E, Reisbach G, Sandberg ML (2003). Latent membrane protein 1 is critical for efficient growth transformation of human B cells by Epstein-Barr virus. Cancer Res.

[pbio-0030404-b30] Humme S, Reisbach G, Feederle R, Delecluse HJ, Bousset K (2003). The EBV nuclear antigen 1 (EBNA1) enhances B cell immortalization several thousandfold. Proc Natl Acad Sci U S A.

[pbio-0030404-b31] Neuhierl B, Feederle R, Hammerschmidt W, Delecluse HJ (2002). Glycoprotein gp110 of Epstein-Barr virus determines viral tropism and efficiency of infection. Proc Natl Acad Sci U S A.

[pbio-0030404-b32] Delecluse HJ, Hilsendegen T, Pich D, Zeidler R, Hammerschmidt W (1998). Propagation and recovery of intact, infectious Epstein-Barr virus from prokaryotic to human cells. Proc Natl Acad Sci U S A.

[pbio-0030404-b33] Frisan T, Levitsky V, Masucci M (2001). Limiting dilution assay. Methods Mol Biol.

[pbio-0030404-b34] Garrone P, Neidhardt EM, Garcia E, Galibert L, van Kooten C (1995). Fas ligation induces apoptosis of CD40-activated human B lymphocytes. J Exp Med.

[pbio-0030404-b35] Zarnegar B, He JQ, Oganesyan G, Hoffmann A, Baltimore D (2004). Unique CD40-mediated biological program in B cell activation requires both type 1 and type 2 NF-kappaB activation pathways. Proc Natl Acad Sci U S A.

[pbio-0030404-b36] Pearson GR, Luka J, Petti L, Sample J, Birkenbach M (1987). Identification of an Epstein-Barr virus early gene encoding a second component of the restricted early antigen complex. Virology.

[pbio-0030404-b37] Kempkes B, Spitkovsky D, Jansen-Durr P, Ellwart JW, Kremmer E (1995). B-cell proliferation and induction of early G1-regulating proteins by Epstein-Barr virus mutants conditional for EBNA2. EMBO J.

[pbio-0030404-b38] Schepers A, Pich D, Hammerschmidt W (1996). Activation of oriLyt, the lytic origin of DNA replication of Epstein-Barr virus, by BZLF1. Virology.

[pbio-0030404-b39] Feederle R, Kost M, Baumann M, Janz A, Drouet E (2000). The Epstein-Barr virus lytic program is controlled by the co-operative functions of two transactivators. EMBO J.

[pbio-0030404-b40] Sinclair A, Palmero I, Peters G, Farrell PJ (1994). EBNA-2 and EBNA-LP cooperate to cause G0 to G1 transition during immortalization of resting human B lymphocytes by Epstein-Barr virus. EMBO J.

[pbio-0030404-b41] Hardwick JM, Bellows DS (2003). Viral versus cellular BCL-2 proteins. Cell Death Differ.

[pbio-0030404-b42] Shen Y, Shenk TE (1995). Viruses and apoptosis. Curr Opin Genet Dev.

[pbio-0030404-b43] Marchini A, Tomkinson B, Cohen JI, Kieff E (1991). BHRF1, the Epstein-Barr virus gene with homology to Bcl2, is dispensable for B-lymphocyte transformation and virus replication. J Virol.

[pbio-0030404-b44] Lee MA, Yates JL (1992). BHRF1 of Epstein-Barr virus, which is homologous to human proto-oncogene bcl2, is not essential for transformation of B cells or for virus replication in vitro. J Virol.

[pbio-0030404-b45] Foghsgaard L, Jaattela M (1997). The ability of BHRF1 to inhibit apoptosis is dependent on stimulus and cell type. J Virol.

[pbio-0030404-b46] Murray PG, Swinnen LJ, Constandinou CM, Pyle JM, Carr TJ (1996). BCL-2 but not its Epstein-Barr virus-encoded homologue, BHRF1, is commonly expressed in posttransplantation lymphoproliferative disorders. Blood.

[pbio-0030404-b47] Flano E, Kim IJ, Woodland DL, Blackman MA (2002). Gamma-herpesvirus latency is preferentially maintained in splenic germinal center and memory B cells. J Exp Med.

[pbio-0030404-b48] Willer DO, Speck SH (2003). Long-term latent murine Gammaherpesvirus 68 infection is preferentially found within the surface immunoglobulin D-negative subset of splenic B cells in vivo. J Virol.

[pbio-0030404-b49] Gibson W, Roizman B (1971). Compartmentalization of spermine and spermidine in the herpes simplex virion. Proc Natl Acad Sci U S A.

[pbio-0030404-b50] Booy FP, Newcomb WW, Trus BL, Brown JC, Baker TS (1991). Liquid-crystalline, phage-like packing of encapsidated DNA in herpes simplex virus. Cell.

[pbio-0030404-b51] Thorley-Lawson DA (2001). Epstein-Barr virus: Exploiting the immune system. Nat Rev Immunol.

[pbio-0030404-b52] Thorley-Lawson DA, Babcock GJ (1999). A model for persistent infection with Epstein-Barr virus: The stealth virus of human B cells. Life Sci.

[pbio-0030404-b53] Küppers R (2002). Molecular biology of Hodgkin's lymphoma. Adv Cancer Res.

[pbio-0030404-b54] Liu YJ, Joshua DE, Williams GT, Smith CA, Gordon J (1989). Mechanism of antigen-driven selection in germinal centres. Nature.

[pbio-0030404-b55] Caldwell RG, Wilson JB, Anderson SJ, Longnecker R (1998). Epstein-Barr virus LMP2A drives B cell development and survival in the absence of normal B cell receptor signals. Immunity.

[pbio-0030404-b56] Mancao C, Altmann M, Jungnickel B, Hammerschmidt W (2005). Rescue of ‘crippled' germinal center B cells from apoptosis by Epstein-Barr virus. Blood.

[pbio-0030404-b57] Neuhierl B, Delecluse HJ (2005). Molecular genetics of DNA viruses: Recombinant virus technology. Methods Mol Biol.

[pbio-0030404-b58] Cherepanov PP, Wackernagel W (1995). Gene disruption in *Escherichia coli* TcR and KmR cassettes with the option of Flp-catalyzed excision of the antibiotic-resistance determinant. Gene.

[pbio-0030404-b59] Posfai G, Koob MD, Kirkpatrick HA, Blattner FR (1997). Versatile insertion plasmids for targeted genome manipulations in bacteria: Isolation, deletion, and rescue of the pathogenicity island LEE of the *Escherichia coli* O157:H7 genome. J Bacteriol.

[pbio-0030404-b60] O'Connor M, Peifer M, Bender W (1989). Construction of large DNA segments in *Escherichia coli*. Science.

[pbio-0030404-b61] Hammerschmidt W, Sugden B (1988). Identification and characterization of oriLyt, a lytic origin of DNA replication of Epstein-Barr virus. Cell.

[pbio-0030404-b62] Takada K, Horinouchi K, Ono Y, Aya T, Osato T (1991). An Epstein-Barr virus-producer line Akata: Establishment of the cell line and analysis of viral DNA. Virus Genes.

